# Healthcare providers’ digital competency: a cross-sectional survey in a low-income country setting

**DOI:** 10.1186/s12913-020-05848-5

**Published:** 2020-11-09

**Authors:** Kirubel Biruk Shiferaw, Binyam Chakilu Tilahun, Berhanu Fikadie Endehabtu

**Affiliations:** 1 Department of Health Informatics, Debre Markos University, Health Science College, Debre Markos, Ethiopia; 2 Department of Health Informatics, University of Gondar, Institute of Public Health, Gondar, Ethiopia

**Keywords:** Digital competency, Digital literacy, Healthcare providers, eHealth, Low income country, Digital health

## Abstract

**Background:**

Healthcare providers across all clinical practice settings are progressively relying and adapting information communication technologies to perform their professional activities. In this era of technology, healthcare providers especially in lower income countries should have at least basic digital competency if a successful application of technology is to be achieved. The aim of this study was to assess digital competency of healthcare providers among seven public health centers in North-West Ethiopia.

**Methods:**

A cross-sectional study design was applied to assess the basic digital competency of healthcare providers working in seven public health centers in North-west Amhara regional state, Ethiopia. Self-administered questionnaire adopted from the European commission’s digital competency framework for assessing digital competency were used. A multivariable logistic regression was performed to identify factors associated with basic digital competency with *p*-value< 0.05 as a rule out for statistical significance. The strength of association was explained in terms of coefficient estimate, adjusted odds ratio and a 95% confidence interval (CI).

**Result:**

From the total of 193 healthcare providers included in the study, 167 of them responded which is a response rate of 86.5%. The majority of respondents 88 (52.7%) were males and the mean age was 28.2 years with a standard deviation of 5.5 years. The result indicated that all items demonstrated an adequate level of internal consistency with Cronbach alpha > 0 .7. Healthcare providers in those public health centers reported that problem solving, safety and communication are the most common challenges encountered. The multivariable logistic regression model indicated that factors such as sex, educational status, profession type, monthly income and years of experience are statistically significant predictors.

**Conclusion:**

Basic digital competency level of healthcare providers working in public health centers in this setting is relatively low. The results highlight the need to improve digital competency among healthcare providers focusing on the identified skill gaps.

## Background

Worldwide, various public and privet sectors are fetching and effectively utilizing simple and advanced technological artifacts to improve their productivity and maintain their competitive advantage [1]. The healthcare sector is one of the potential application areas where advanced technologies could improve health service and health system as a whole [2–4]. The healthcare sector has been known for its slower adoption of new technologies [5, 6] and it is even slower in middle and low income countries [7–9]. However, health care professionals across all clinical practice settings are progressively relying and adapting information communication technologies to perform their professional activities [10–13]. Several studies have identified the need for a certain level of digital competency in order to make an efficient and effective use of technologies among different allied health professions [14–16]. Digital competency of healthcare providers could greatly help the adoption of Electronic Medical Record systems (EMRs) and online risk assessment and decision support tools, as well as the introduction of cutting-edge medical equipment which often have digital user interfaces.

The concept of digital literacy/competency is also known as digital information literacy. Digital information literacy is defined by the European Union Commission as “*the set of knowledge, skills, attitudes, abilities, strategies, and awareness that are required when using ICT and digital media to perform tasks; solve problems; communicate; manage information; collaborate; create and share content; and build knowledge effectively, efficiently, appropriately, critically, creatively, autonomously, flexibly, ethically, reflectively for work, leisure, participation, learning, and socializing*” [17, 18].

In this era of technology, healthcare professionals particularly in low-income countries should have at least a minimum level of digital competency if technology is to be applied successfully. Studies have identified the need to bridge the digital skill gap of healthcare providers in order to transfer technology to the point where health service quality is maintained. However, most of these studies do not specify in detail where the critical digital skill gap lays [19–21]. By itself, digital literacy is a congregating and evolving concept through time and advancement in technology. Thus, *Martin (2006)* argues the need to maintain up to date level of digital skill. *Martin (2006)* asserted that digital literacy is not just a threshold one could achieve through certification and diploma at a point time. Rather, it is a temporary and context based concept referring only to the current level of performance [22]. As a result, assessing the level of digital competency should be conducted in parallel with the evolving changes in the digital environment.

In Ethiopia, the government has implemented a strategy that focuses on digitalizing the health system and currently, there are many eHealth project initiatives underway and most of these initiatives are faced with the challenge of sustainability. This could be the result of different factors including a low level of digital competency among target users [23–25]. To bridge this gap, the commonly proposed solution is to provide computer training for healthcare providers before or during the implementation of computer-based systems. However, we argue that offering general computer training might not be sufficient to bridge the digital competency gap. Based on the new system’s requirement, one has to assess the proficiency level of user’s digital literacy first. Low productivity, inefficiency and missed opportunities are the key consequences and real-world implications of low digital competency. This could mean medical information is lost or recorded incorrectly, technologies could pass by unadopted, general healthcare standards could fall behind contemporary standards, confidential patient information could be accessed by unauthorized parties, etc. According to the European commission’s classification, digital users could be categorized as basic, intermediate and advanced users. Based on the technology’s enquiry of digital skill level, one should assess the required digital literacy level of potential users and identify the core gaps which needs more attention. In such a way, one might have a clear understanding of the target population. Several studies pined the need to assess digital competency level of potential digital device users in order to give the appropriate level of education and training [26–28].

The European Commission has developed a digital competency framework to elucidate the context and aspects of digital competency. The framework identified five core digital literacy components (*Information/data literacy, content creation, communication, problem solving and safety*) to describe and understand the digital competency level of individuals [17]. To ensure a successful digitization of the health system in Ethiopia and other African countries, understanding the level of healthcare providers’ digital competency could be considered baseline information. The aim of this study was to assess the basic digital competency level of healthcare providers among seven public health centers in North-West Ethiopia.

## Methods

### Study design and setting

A cross-sectional study design was applied to assess basic digital competency level of healthcare providers working in seven public health centers in North-west Amhara regional state, Ethiopia. Health centers are the smallest healthcare service delivery units next to health posts, which were established to improve healthcare coverage. There were approximately 4212 health centers and 18,151 health posts in Ethiopia when this study was conducted. The health centers were selected based on the priority list of health centers in which the Federal Minister of Health (FMoH) and agencies planned to introduce electronic interventions such as EMR (Electronic Medical Record) systems or mHealth (Mobile Health) interventions. The Ethiopian FMoH have a strategic plan to substantially advance five major types of health information systems; Tele-education and Telemedicine, EMR/EHR (Electronic Medical Record/ Electronic Health Record), mHealth, eCHIS (electronic Community Health Information System) and eLMIS (Electronic Logistics Management Information System). This study is focused on the context of Electronic Medical Record systems (EMRs) in health facilities.

### Sample size and participants

The health facilities have approximately 193 healthcare providers in total and all healthcare providers who are directly engaged in both clinical and non-clinical health service delivery were considered for the study. Since the total number of potential participants is relatively small, all healthcare providers were approached for data collection. Healthcare providers with annual and sick leave were excluded.

### Outcome and outcome measure

The outcome variable is digital competency/literacy and it was measured by twenty-two items divided in to five major components [17]. All items were measured in 5-point Likert scale ranging from strongly agree (5) to strongly disagree (1). The first component was information processing. Information processing is an important element in understanding the competency level of participants focused majorly on individual’s ability to search, find, appraise, sort, store and retrieve information using digital devices. The second component is Content creation*.* Content creation describes an individual’s ability to create/delete /manipulate contents such as text and images in different application software such as Microsoft Word and Excel in digital devices. It also includes adjusting settings based on one’s interest of use. On the other hand, communication focuses on an individual’s capability to communicate, share and interact with others using digital devices and network. It includes internet or local area connections. Problem solving focuses on assessing the skill of individual’s potential in solving routine hardware and software problems encountered while using digital devices. Problem solving also evaluates where a person stops working when difficulties appear or they look for digital solutions. The last component is Safety, which assesses what people do to protect their devices from cyber/physical attack and the precautions they take on their own health. For this study, we used a paper-based self-administered questionnaire adopted from European commission’s digital competency framework for assessing digital competency [17]. Due to the non-normal distribution of outcome variable, scores less than the median value were labelled ‘low digital competency’, while scores greater-than-or-equal-to the median value were labelled ‘high digital competency’. Internal consistency of items was assessed using Cronbach alpha. The tool was adopted the European commission’s digital competency framework and pretested on 30 [29] healthcare providers in Debremarkos health center for its content validity and readability (Kirubel Tool). Data collectors were recruited and trained to maintain the data quality and continuous supervision was performed by the investigators during the study period.

### Quality control & analysis methods

Data collectors were given half a day training on correct participant approach and data handling. Data collectors were supervised during the data collection period. Following this, data were checked for completeness and accuracy, then entered into the Statistical Package for Social Science (SPSS version 23) for further analysis. Descriptive statistics were calculated to summarize healthcare providers’ socio-demographic characteristics. Before running the multivariable logistic regression, assumptions for multicollinearity, outliers and independent error terms were tested. Multicollinearity was verified by running a pseudo linear regression repeating the independent variables as an outcome variable. The results confirmed the absence of multicollinearity with a variance inflation factor (VIF) value less than three and tolerance greater than 0.7 [30]. No significant outlier effect was observed in the box plot. Goodness of model fit was tested using omnibus test for global fitness; the Hosmer and Lemeshow test was applied to examine fitness of the data to the model. Accordingly, the omnibus test result was significant with *p*-value < 0.05 and the Hosmer and Lemeshow test shows a good model fit with *p-*value = 0.712 indicating good model fitness [31]. A multivariable logistic regression was applied to identify factors associated with basic digital competency with *p*-value< 0.05. The strength of association was explained in terms of coefficient estimate, adjusted odds ratio and a 95% confidence interval (CI).

## Result

From the total of 193 healthcare providers included in the study, 86.5% (167) of them responded to the invitation to participate. The majority of respondents 88 (52.7%) were males and the mean age was 28.2 years with a standard deviation of 5.5 years. Diploma holders were relatively larger in number with 93 (55.7%) and most of the study participants were nurses with 83 (90.7%). The average work experience of participants were 4.6 years with a standard deviation of 3.9 years. Table 1 summarizes the socio-demographic characteristics of study participants.

**Table 1:** Socio- demographic characteristics of participants

Socio-demographic characteristics	Number	Percent
Sex		
Male	**88**	**52.7**
Female	**79**	**47.3**
Educational Status		
Diploma	**93**	**55.7**
Degree	**71**	**42.5**
Master	**3**	**1.8**
Profession type		
Nurse	**83**	**49.7**
Health Officer	**13**	**7.8**
Medical laboratory	**13**	**7.8**
Midwifery	**34**	**20.4**
Pharmacy	**24**	**14.4**
Monthly Income^a^		
1500–3500	**45**	**26.7**
3501–5500	**107**	**64.1**
> 5500	**15**	**9.0**

^a^*The currency of Monthly income is in Ethiopian birr (ETB)*

The result indicated that all items demonstrated an adequate level of internal consistency with Cronbach alpha (α > 0.7) [32]. One can score minimum of 22 and maximum of 110 points from the digital competency items. Almost half of the participants (49.7%) reported that they have high digital competency. From the components of digital competency, problem solving, safety and communication were the lowest rated with 73.1, 63.5 and 58.7% reporting a low level of competency respectively (See Table 2).

**Table 2:** Components of digital competency

Components	Low		High		Mean	Standard deviation
	Frequency	%	Frequency	%		
Information processing	83	50.3	84	49.7	10.0	3.3
Content creating	87	52.1	80	47.9	16.7	5.2
Communication	98	58.7	69	41.3	12.8	3.8
Safety	106	63.5	61	36.5	16.1	4.8
Problem solving	122	73.1	45	26.9	15.3	5.0

**Fig. 1:**
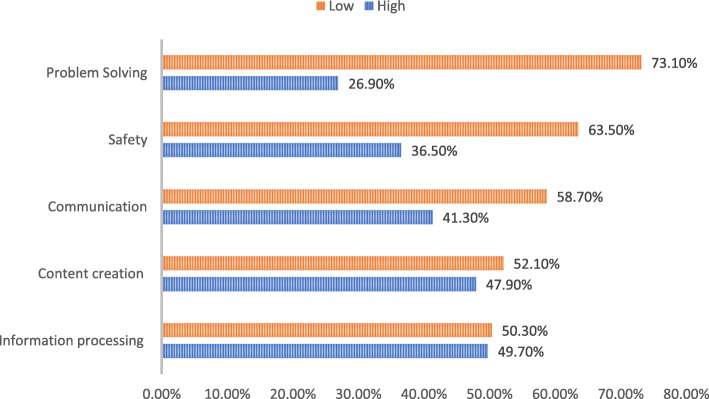
Digital competency composition

Figure 1 shows that healthcare providers reported that problem solving (solving routine hardware and software problems encountered while using digital devices/systems), safety (ability to take safety measures in regard with the user and the devices/systems) and communication (ability to communicate, share and interact with others using digital devices/systems) are the most common challenges encountered. In contrast, information processing (ability to search, find, appraise, sort, store and retrieve information using digital devices) and content creation skills (ability to create/delete /manipulate contents such as text and images) are relatively easier tasks. (see Fig. 1).

The multivariable logistic regression model indicated that factors such as sex, educational status, profession type, monthly income and year of experience are statistically significant predictors of healthcare providers’ digital competency in this setting. The results also indicated that medical laboratory professionals and pharmacists demonstrated relatively lower digital competency level compared to nurses. Higher monthly income and increased years of experience are also associated with lower digital competency. (see Table 3).

**Table 3:** Multivariable logistic regression results

Variables	Coefficient estimate	AOR	95% CI		*p*-value
			Lower	Upper	
Sex					
Male	1.365	3.914	1.745	8.776	.001^a^
Female					
Age	.105	1.111	.924	1.336	.262
Educational status					
Diploma and below	−1.004	.366	.149	.900	.029^a^
Degree and above					
Profession Type					.004^a^
Health officer	−.694	.499	.198	1.263	.142
Medical laboratory	−3.046	.048	.005	.423	.006^a^
Pharmacy	−2.437	.087	.013	.600	.013^a^
Midwifery	.531	1.700	.476	6.076	.414
Nurse					
Monthly Income					.005^a^
1500–3500					
3501–5500	−2.251	.105	.012	.923	.042^a^
> 5500	−2.931	.053	.008	.372	.003^a^
Year of Experience	−.372	.689	.518	.918	.011^a^

Reference group: female, degree and above, nurse, 1500–3500, ^a^indicates statistical significance, the currency of monthly income is in Ethiopian birr (ETB), *AOR* adjusted odds ratio

## Discussion

The findings from this study suggest that healthcare providers working in health centers lack problem solving, safety and communication competencies in using digital devices. Sex, educational status, profession type, monthly income and years of experience were statistically significant factors that influenced participants’ overall level of digital competency.

To the authors’ knowledge, this is the first study to assess basic digital competency level of healthcare providers that uses the European commission’s digital competency framework.

The increasing prominence of information communication technologies (ICTs) in healthcare setting has prompted numerous studies to identify bottlenecks and discover means to successfully implement ICT in the healthcare settings [33, 34]. Studies outlined the need to build staff capacity in different ways including the provision of continuous and focused training for healthcare workers [35, 36]. Participants in this study demonstrated relatively low basic digital competency with majority of them reporting a lack of basic technical skills to solve routine hardware and software problems. This finding is similar to previous studies conducted in different settings (Scotland, Maryland) [15, 21, 37] and in contrast with other studies reported higher digital competency of participants [38]. The difference could be due to difference in study setting where the staff composition and digital facilities in health centers are limited compared to tertiary and secondary hospitals. The finding implies that training focused on improving routine problem solving skills, communication, and safety measures could significantly improve the overall digital competency of healthcare workers. Unlike studies that confirm the significance of age difference in predicting participants’ digital competency level [39, 40], this study found that there is no significant association between participants’ age and digital competency level. The possible explanation for this difference could be due to the fact that there is no significant generation gap in age distribution among participants in this study. On the other hand, sex was a significant variable in this study indicating male participants are more likely to possess higher digital competency with (AOR: 3.9 CI: 1.7–8.8). This finding is different from studies conducted elsewhere [41, 42] and in line with other studies [43, 44]. The possible reason for this disparity could be due to the fact that digital divide and gender inclusion is still a prominent challenge for middle- and lower-income countries such as Ethiopia. The finding also indicated that participants with an educational status of diploma and below are 63.4% less likely to possess high digital competency. This is consistent with other studies which report a positive relationship between higher education levels and high digital competency [21, 43]. Profession type was also a predictive variable. Medical laboratory professionals and pharmacists demonstrated lower digital competency with 95.2 and 91.3% less likely to possess high digital competency level respectively. This finding is in line with other studies confirming that pharmacists lack digital competency [45, 46]. This implies that although some allied health professions are not directly engaged in the clinical care provision, their inability to work hand in hand with digital technology influences the overall success of technology adoption in the health sector. Therefore, providing tailored digital education for allied health professionals working in both clinical and non-clinical environments could improve the overall digital competency level of healthcare providers which in turn increases the likelihood of successful implementation of ICT in healthcare system. An increase in monthly income and years of experience are associated with lower digital competency. Participants with monthly income greater than 5500 ETB are 94.7% less likely to possess higher digital competency level with CI: (0.008,0.372). A one unit increase in years of experience results in 31% less likelihood of possessing higher digital competency. In this study setting, an increase in income is associated with an increase in years of experience, where promotions are typically based on years of experience. This finding is similar to other studies indicating longer years of experience is associated with lower digital competency [21, 36, 46]. The possible reason could be that younger professionals are more receptive and adaptable to changes in the working environment compared to older ones. In almost all lower and lower middle income countries, the healthcare system is structured as an in-person or face-to-face model of care, and following the 2020 COVID-19 pandemics, the usual health care service has been altered significantly [47]. Although there is no clear evidence demonstrating a mismatch between patients and healthcare providers regarding their communication preferences, the global death toll resulting from COVID-19 has indicated the need to strengthen the digital capacity of health professionals and health services.

The present study is limited by a relatively small sample size. A larger group of representative samples from all health centers in Ethiopia would be more generalizable to the wider study population. It would also be more informative for decision makers if future research investigates basic, intermediate, and proficient levels of digital competency across a range of health professions.

## Conclusion

This study found that sex, educational status, profession type, monthly income and years of experience all significantly impact healthcare providers’ digital competency. Problem solving, communication and safety are the main reasons for lower scores in digital competency. The overall digital competency level of healthcare providers in our sample of north-west Ethiopia is relatively low. There is a clear need to improve digital competency in healthcare providers working in health centers in Ethiopia.

## Data Availability

The datasets used and/or analyzed during the current study is available from the corresponding author on reasonable request.

## Abbreviations

AOR: Adjusted Odds Ratio
CI: Confidence Interval
SPSS: Statistical Package for Social Science
VIF: Variance Inflation Factor
FMoH: Federal Minister of Health
EMR: Electronic Health Record
mHealth: Mobile Health
eCHIS: Electronic Community Health Information System
eLMIS: Electronic Logistics Management Information System
